# Marital Quality as a Moderator of the Association Between Sensory Impairments and Cognitive Functioning

**DOI:** 10.1093/geroni/igab046.1468

**Published:** 2021-12-17

**Authors:** Jeremy Yorgason, Corinna Tanner, Avalon White, Stephanie Richardson, Melanie Hill, Shaylee Bench, Brian Stagg, Joshua Ehrlich

**Affiliations:** 1 Brigham Young University, Provo, Utah, United States; 2 College of Nursing, Brigham Young University/Provo, Utah, United States; 3 School of Family Life, Brigham Young University/Provo, Utah, United States; 4 University of Utah Moran Eye Center, University of Utah/Salt Lake City, Utah, United States; 5 University of Michigan, University of Michigan, Michigan, United States

## Abstract

Research suggests that marital quality may buffer the impact of sensory impairments in later life, and that marital quality relates to cognitive functioning. This study explored how marital quality moderated links between sensory impairments and cognitive functioning. We used data from 723 paired marital dyads from two cohorts in the NHATS and NSOC studies across three-year periods (n=340 dyads from waves 1, 2, 3; n=383 dyads from waves 5, 6, 7). Growth curve models of executive functioning indicated that marital quality moderated effects of both hearing and vision impairment on changes in cognitive functioning longitudinally. Specifically, higher marital quality was associated with higher executive functioning across time. Results suggested no improvement in executive functioning among those with average or lower marital quality. Although cognition declines with advanced age and with sensory impairments, results suggest that older adults with higher marital quality may improve in some aspects of cognition longitudinally.

